# Cardiac Adverse Events in Patients Receiving Immune Checkpoint Inhibitors in the Adjuvant Setting: An FDA Pooled Analysis

**DOI:** 10.1111/anec.70087

**Published:** 2025-05-07

**Authors:** Asma Dilawari, Mori J. Krantz, Ilynn Bulatao, Hee‐Koung Joeng, Marc Neilson, Suparna Wedam, Xin Gao, Mallorie H. Fiero, Abhilasha Nair, Marc Theoret, Laleh Amiri‐Kordestani

**Affiliations:** ^1^ Center for Drug Evaluation and Research (CDER) U.S. Food and Drug Administration Silver Spring Maryland USA; ^2^ Oncology Center of Excellence (OCE) U.S. Food and Drug Administration Silver Spring Maryland USA

**Keywords:** adjuvant, cancer, cardio oncology, cardiotoxicity, immune checkpoint inhibitors, immunotherapy

## Abstract

**Background:**

Immune checkpoint inhibitors (ICIs) have revolutionized cancer treatment. By releasing blocks (checkpoints) on the immune system, they elicit powerful antitumor effects. Despite improving survival, ICIs are associated with serious cardiac toxicities. Previous reports have focused on advanced cancer; cardiotoxicity data are therefore limited in the curative setting. We evaluated ICI cardiotoxicity in the non‐metastatic setting, where long‐term cardiac safety is a growing public health concern.

**Methods:**

ICIs approved in the adjuvant setting were pooled and trials with combination chemotherapy were excluded. Cardiac adverse events (AEs) and emerging cardio‐metabolic risks (hyperglycemia, weight gain, hypothyroidism) were assessed. The relative risk (RR) of cardiotoxicity was assessed.

**Results:**

Ten randomized controlled trials of atezolizumab, ipilimumab, nivolumab, and pembrolizumab in multiple solid tumors were evaluated; among 9244 patients, 5338 received ICIs. No trial performed routine cardiac monitoring. Six percent of ICI patients vs. 4.6% in placebo (RR 1.24, 95% CI 1.04, 1.49) had a cardiac AE and 13 (0.2%) of ICI patients experienced a fatal cardiac AE (RR 4.76, 95% CI 1.07, 21.06). Older age and male sex were associated with a higher risk for cardiac fatality. Arrhythmia was the most common cardiac AE; hypothyroidism was more frequent (14% vs. 2.5%) among ICI‐treated patients.

**Conclusion:**

This is the largest pooled analysis of cardiac AEs associated with ICIs in the adjuvant setting. Despite no formalized testing for subclinical cardiotoxicity, ICI treatment increased cardiac AEs. These findings are relevant for long‐term cancer survivors, clinicians, and particularly in new drug development, where cardiotoxicity may be substantially underestimated.

## Graphical abstract

ICI treatment impacts different areas of the heart and cardiac function. There are multiple potential mechanisms for ICI related cardiotoxicities. Inflammatory cytokines and T‐lymphocyte infiltration may cause necrosis and cellular injury in the myocardium. PD‐1 expression on myocytes may lead to cross‐reactivity with tumors and the myocardium. Inhibition of PD‐1/PD‐L1 could remove the protection against immune‐related damage to cardiomyocytes. ICIs have been reported to increase oxidative stress, the risk of hypertension, and atherosclerosis. Electrocardiogram (ECG), echocardiogram (ECHO), computed tomography (CT), magnetic resonance imaging (MRI), and angiogram abnormalities may be used to diagnose these toxicities.

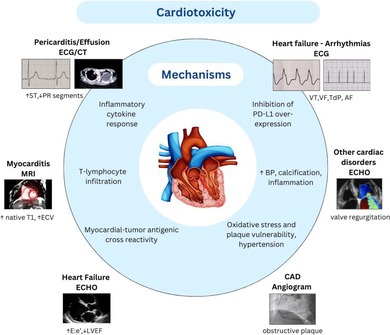

**Abbreviations**
AE: adverse event
CI: confidence interval
CTCAE: common terminology criteria for adverse events
HR: hazard ratio
MedDRA: Medical Dictionary for Regulatory Activities
NCI: National Cancer Institute
NCT: National Clinical Trial number
NT‐BNP: N‐terminal pro‐brain natriuretic peptide
RR: relative risk
USPI: United States Prescribing Information

## 1 Introduction

Cardiovascular disease (CVD) and cancer are the two leading causes of death globally. CVD affects almost half of U.S. adults, and the number of cancer survivors in the U.S. is projected to reach 20 million (Tsao et al. 2023; Siegel et al. 2024). Higher rates of CVD in cancer survivors and increased diagnoses of cancer in people with heart disease have both been reported, demonstrating important links between these two conditions (van den Berg et al. 2024; Armenian et al. 2018). While the field of cardio‐oncology has expanded in the last decade, the dramatic growth in new therapies in oncology continues to create knowledge gaps. Specifically, the type and extent of cardiac toxicities associated with immune checkpoint inhibitors (ICIs) is critical since this class has now been approved for use in early‐stage, curable cancers.

ICIs work by releasing blocks (checkpoints) on the body's immune response, thereby enhancing the body's ability to kill tumor cells. ICIs have been approved for multiple indications by the US Food and Drug administration (FDA), and their development has been heralded as a revolutionary milestone in oncology (Ascierto et al. 2020; Eggermont et al. 2015; Hodi et al. 2010; Kelly et al. 2021; Sharma et al. 2021; Bajorin et al. 2021) Since the first FDA approval of ipilimumab in 2011, ICIs have been used to treat a spectrum of malignancies, including melanoma, non‐small cell lung, head and neck, and gastrointestinal cancers (Darvin et al. 2018; Hodi et al. 2010). As with most cancer therapies, the safety and efficacy of ICIs were initially evaluated in advanced, refractory, or incurable cancer. After these successes, trials explored the use of ICIs to prevent recurrence in patients with early‐stage malignancies being treated with curative intent. From 2015 to 2023, four ICIs were FDA‐approved as monotherapy in the adjuvant setting to prevent cancer recurrence, in some cases nearly doubling rates of disease‐free survival (Eggermont et al. 2015; Hodi et al. 2010; Kelly et al. 2021; Sharma et al. 2021; Bajorin et al. 2021). Yet, this efficacy must be considered in the context of emerging data on cardiotoxicity. ICI‐associated myocarditis (Thuny et al. 2022; Patel et al. 2023; Piazza et al. 2025), and reports of arrhythmias, coronary artery disease, and heart failure were prominent signals, yet likely underreported in early trials (Sharma et al. 2024). Although the mechanism of ICI‐associated cardiac toxicity is not established, multiple effects on cardiac structure occur (Central Illustration) (Rubio‐Infante et al. 2021; Li et al. 2025). With the increasing use of ICIs in curative settings, understanding the long‐term cardiac impact on cancer survivors necessitates new approaches to evaluating clinical trial data. Ensuring that trial data captures cardiac toxicity is imperative as this informs drug labeling, impacts providers' clinical practice, and ensures proper monitoring posttreatment.

The myriad effects of immunotherapy on cardiovascular health make it challenging to measure toxicity within the confines of a single clinical trial. While previous meta‐analyses have reported trial‐level data from public databases, the predominant focus was advanced, metastatic cancers and often combined randomized and observational trials together. Given this background, we performed a pooled analysis of cardiovascular adverse events in 10 clinical trials that supported FDA approval of ICIs in early stage, curable cancer.

## 2 Methods

Patient level data was pooled from 10 randomized controlled trials submitted to the FDA to support approval of 4 different ICIs in 10 adjuvant solid tumor indications between October 2015 and October 2023 (Table 1). Single‐arm trials and those with combination ICI plus chemotherapy treatment arms were excluded. Ten randomized trials met these criteria (Table 1). Since these trials have been submitted to the FDA, full data access and deidentification obviated requirements for external permissions to conduct research. Cardiac adverse events (AEs) were identified based on high‐level Medical Dictionary for Regulatory Activities (MedDRA) preferred terms under the Cardiac Disorders System Organ Class (arrhythmias, palpitations, coronary artery disease, myocarditis, cardiac failure, pericardial effusion, other cardiac disorders). Additional adverse events categorized as cardiac risk factors with their associated preferred terms were also evaluated in a descriptive analysis (thyroid disorders, weight gain, lipid disorders, metabolic disorders). CTCAE, a standardized grading system developed by the National Cancer Institute (NCI) to help evaluate adverse events associated with new cancer therapies, was used to categorize the severity of these AEs with higher Grade 3–4 toxicities being more severe and Grade 5 being fatal.

**TABLE 1:** Clinical trials of adjuvant ICI treatment.

Drug	Comparator	Disease site	Trial	Disease‐free survival/relapse‐free survival (months)	Hazard ratio (95% CI)
Atezolizumab	Best supportive care^a^	NSCLC	NCT02486718	42.3 vs. 35.3	0.66 (0.50, 0.88)
Ipilimumab	Placebo	Melanoma	NCT00636168	26.1vs. 17.1	0.75 (0.64, 0.90)
Nivolumab	Ipilimumab	Melanoma	NCT02388906^b^	NR	0.65 (0.53, 0.80)
Nivolumab	Placebo	Urothelial cancer	NCT02632409	20.8 vs. 10.8	0.70 (0.57, 0.86)
Nivolumab	Placebo	Esophageal cancer	NCT02743494	22.4 vs. 11	0.69 (0.56, 0.85)
Nivolumab	Placebo	Melanoma	NCT04099251	NR	0.42 (0.30, 0.59)
Pembrolizumab	Placebo	NSCLC	NCT02504372^c^	53.6 vs. 42	0.76 (0.63, 0.91)
Pembrolizumab	Placebo	Renal cell carcinoma	NCT03142334	NR	0.68 (0.53, 0.87)
Pembrolizumab	Placebo	Melanoma	NCT02362594	NR vs. 20.4	0.57 (0.46, 0.70)
Pembrolizumab	Placebo	Melanoma	NCT03553836	NR	0.65 (0.46, 0.92)

*Note:* Ten trials supporting approvals of monotherapy ICI treatment in the adjuvant setting are listed with the respective comparator arms, malignant diagnosis, and efficacy results (disease‐free or recurrence‐free survival) reflected in the USPI and/or published results. FDA granted approvals for these indications from 2015 to 2023.

Abbreviations: CI, confidence interval; NCT, National Clinical Trial number; NSCLC, non‐small cell lung cancer; NR, not reached; USPI, United States Prescribing Information.

^a^ Best Supportive care included observation and serial imaging.

^b^ For NCT02388906, a study comparing two ICIs (nivolumab vs. ipilimumab), patients in both arms were included in the ICI group.

^c^ Results of this trial are efficacy analyses from published results, not USPI, to reflect results of the overall population (Ellison and Nohria 2023).

Treatment‐emergent cardiac adverse events and TEAEs related to cardiovascular risk experienced by patients were summarized using percentage for the patients who received an ICI and the patients who did not, respectively. Relative risk with corresponding 95% CI for experiencing any cardiac AE or death due to cardiac AE in the ICI group versus the non‐ICI was estimated. Among patients who received ICI treatment, the relative risk of cardiac AEs or death by age group (≥ 65 years vs. < 65 years) and by sex (male vs. female) was assessed. These analyses were exploratory; no hypothesis testing was performed. Statistical analyses were conducted using SAS (version 9.4).

## 3 Results

Between 2015 and 2023, a total of 9244 adult patients within 10 randomized controlled studies were identified supporting approval of 4 different ICIs for adjuvant solid tumor indications. (Table 1). Among these, 5338 patients received an ICI, and 3906 patients were treated in the non‐ICI arms. The majority of trials (*n* = 8, 80%) were designed to compare ICI to placebo. One compared ICI to best supportive care/observation (NCT02486718), and another (NCT02388906) compared two ICIs, ipilimumab and nivolumab. Efficacy results are summarized in Table 1, where all studies demonstrated reductions in cancer relapse (hazard ratio range 0.42–0.70). Sociodemographic characteristics of the patients are described in Table 2. Most patients were male, White, < 65 years of age, and enrolled from sites outside the U.S.

**TABLE 2:** Demographic characteristics.

	ICI, *N* = 5338, *N* (%)	Comparator, *N* = 3906, *N* (%)
Gender		
Male	3532 (66)	2617 (67)
Female	1806 (34)	1289 (33)
Age (years)		
Mean (SD)	58 (12)	59 (12)
Median (min—max)	60 (16–92)	61 (17–92)
Age group (years)		
< 65	3502 (66)	2440 (62)
> = 65	1836 (34)	1466 (38)
Race		
Asian	507 (9)	402 (10)
American Indian or Alaska Native	13 (0.2)	4 (0.1)
Black or African American	25 (0.5)	19 (0.5)
Native Hawaiian or other Pacific Islander	3 (0.1)	1 (0.0)
Other^a^	44 (0.8)	22 (0.6)
Unknown/Not Reported	621 (12)	606 (16)
White	4125 (77)	2852 (73)
Region		
Europe	3481 (65)	2624 (67)
USA	884 (17)	532 (14)
Asia	517 (10)	412 (11)
Rest of the world	456 (9)	338 (9)

*Note:* Demographic criteria for patients treated with ICI or in the comparator arm for 10 trials (Table 1) leading to FDA approvals for adjuvant ICI monotherapy to treat solid tumors.

^a^ Race data for certain countries were not allowed to be collected per local regulations and were recorded as “other”.

**FIGURE 1:**
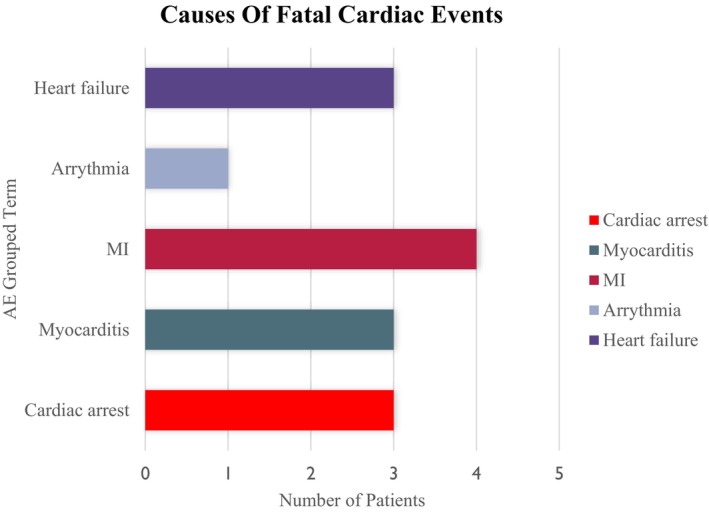
Fatal cardiac adverse events in patients receiving ICIs. Thirteen patients in the FDA pooled analysis receiving ICIs (0.2%) experienced Grade 5 (fatal) cardiac AEs compared to 2 patients in the comparator arm (not pictured). Of these, *n* = 3 experienced cardiac arrest, *n* = 4 experienced an MI (myocardial infarction or myocardial ischemia), *n* = 3 myocarditis, *n* = 3 had heart failure (cardiac failure acute, cardiogenic shock, or cardiopulmonary failure), and 1 had a fatal arrhythmia.

**FIGURE 2:**
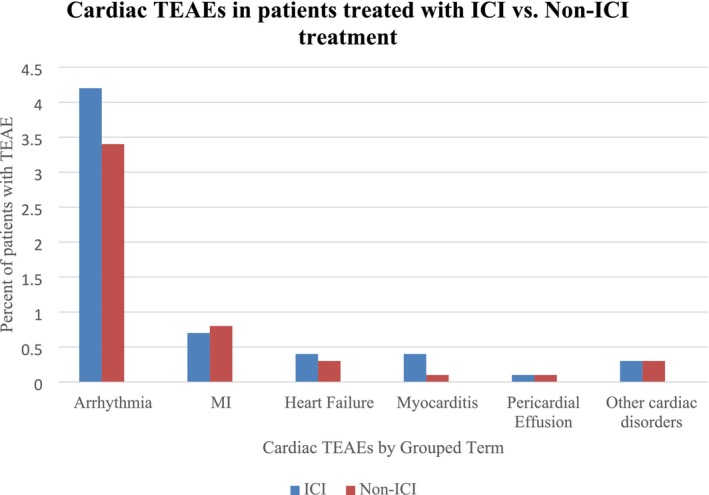
Cardiac treatment emergent events. Using grouped terms, the % of all‐grade cardiac toxicities experienced by patients treated with ICIs vs. non‐ICI treatments in the adjuvant setting is shown. Six percent of patients (*N* = 304) treated with ICIs experienced cardiac TEAEs vs. 4.6% (*N* = 179) treated with non‐ICI therapies. Of these, the most common was arrhythmia. The terms arrhythmia, MI (including coronary artery disease), heart failure, myocarditis, pericardial effusion, and other cardiac disorders were GTs. Arrhythmia includes arrhythmia, tachycardia, bradycardia, extrasystoles, tachyarrhythmia. MI includes myocardial infarction, myocardial ischemia, silent MI, coronary artery stenosis, cardiac arrest, acute coronary syndrome, coronary artery disease: angina pectoris, arteriosclerosis coronary artery, coronary artery stenosis. Heart failure includes cardiac failure, cardiac failure congestive, cardiac failure chronic, cardiopulmonary failure, systolic dysfunction, left ventricular dysfunction, acute left ventricular failure. Myocarditis includes myocarditis, autoimmune pericarditis, immune‐mediated myocarditis, autoimmune myocarditis. Pericardial effusion includes pericardial effusion, cardiac tamponade. Other cardiac disorders included mitral valve incompetence, cardiac disorder, cardiovascular disorder, cardiovascular insufficiency, atrial enlargement, aortic valve incompetence, pericardial cyst, pleuro‐pericarditis, cardiogenic shock, cardiac septal hypertrophy, mitral valve prolapse, mitral valve incompetence, intracardiac thrombus, left ventricular enlargement.

There were 15 deaths due to cardiac AEs across trials, 13 (0.2%) in the ICI group (Figure 1) and 2 (0.1%) in the non‐ICI group. Cardiac AE deaths were attributed to cardiac arrest, myocardial ischemia/infarction, arrhythmia, heart failure, or myocarditis. Six percent of patients receiving ICIs experienced a cardiac AE vs. 4.6% of patients in the non‐ICI group, the most common being arrhythmias. Grade ≥ 3 cardiac AEs were reported in 1.3% of patients receiving ICIs and 1.1% of patients in the non‐ICI group (Figure 2).

Additional AEs linked to the development of CVD included thyroid disease, weight gain, and metabolic disorders. These were assessed given their known impact on future risk for CVD (Table 3). Nineteen percent of patients receiving ICIs reported thyroid dysfunction AEs compared to 3.5% of patients in the non‐ICI arms. The most common thyroid AE reported with ICI use was hypothyroidism (13%); thyroiditis, a known immune‐related risk of ICIs, was reported in 1.6% of patients receiving ICIs. Weight gain, hypertension, hyperglycemia, or hyperlipidemia in patients receiving ICIs versus non‐ICI treated patients did not differ.

**TABLE 3:** Cardio‐metabolic risk in ICI vs. comparator patients.

	ICI, *N* = 5338		Comparator *N* = 3906	
	All grades *n* (%)	Grades 3–4 *n* (%)	All grades *n* (%)	Grades 3–4 *n* (%)
All thyroid‐related AEs	1019 (19)	21 (0.4)	136 (3.5)	0 (0.0)
Hypothyroidism	724 (14)	6 (0.1)	99 (2.5)	0 (0.0)
Hyperthyroidism	451 (8)	10 (0.2)	38 (1.0)	0 (0.0)
Thyroiditis	86 (1.6)	5 (0.1)	5 (0.1)	0 (0.0)
Other thyroid disorder	12 (0.2)	0 (0.0)	1 (0.0)	0 (0.0)
Weight gain^a^	338 (6)	10 (0.2)	419 (11)	12 (0.3)
Hyperglycemia^b^	199 (3.7)	36 (0.7)	164 (4.2)	19 (0.5)
Hyperlipidemia^c^	75 (1.4)	6 (0.1)	54 (1.4)	7 (0.2)
Diabetes mellitus^d^	54 (1.0)	24 (0.4)	25 (0.6)	5 (0.1)

*Note:* Treatment emergent adverse events (TEAEs) were classified by grouped terms which included thyroid disorders, weight gain, hyperglycemia, hyperlipidemia, and diabetes mellitus for patients treated with ICI and non‐ICI comparators in the trials noted in Table 1. TEAEs were separated by severity as per CTCAE v 5.0 criteria.

^a^ Weight gain included weight increased, weight gain.

^b^ Hyperlipidemia included blood cholesterol increased, hypercholesterolemia, blood tryglyceride(s) increased, hyperlipidemia, hypertriglyceridemia, dyslipidemia.

^c^ Hyperglycemia included blood glucose increased, hyperglycemia.

^d^ Diabetes mellitus included diabetes mellitus, type 1 diabetes mellitus, and type 2 diabetes mellitus.

Relative risk (RR) of cardiac AEs by age (< 65 vs. ≥ 65 years) and sex (male vs. female) is shown in Table 4. Patients treated with ICIs who were ≥ 65 years of age had a higher RR of developing any grade cardiac AE (1.28, 95% CI 1.02, 1.60). Male patients compared to female patients and patients age ≥ 65 years compared to patients age < 65 had a higher RR of death due to cardiac AE, with RR 2.81 (95% CI 0.62, 12.67) and 3.05 (95% CI 1.00, 9.32) respectively.

**TABLE 4:** Relative risk of cardiac adverse events: adjuvant setting for ICIs.

	ICIs	
	All grade, *n*/*N* (%)	Grade 5, *n*/*N* (%)
Total AEs	304/5338 (5.7%)	13/5338 (0.2%)
Relative risk (95% CI)	1.24 (1.04, 1.49)	4.76 (1.07, 21.1)
Age (years)		
≥ 65	122/1836 (6.6%)	8/1836 (0.4%)
< 65	182/3502 (5.2%)	5/3502 (0.1%)
Relative risk (95% CI)	1.28 (1.02, 1.60)	3.05 (1.00, 9.32)
Sex		
Male	190/3532 (5.4%)	11/3532 (0.3%)
Female	114/1806 (6.3%)	2/1806 (0.1%)
Relative risk (95% CI)	0.85 (0.68, 1.07)	2.81 (0.62, 12.67)

*Note:* Relative risk of experiencing all Grade (1–5) or Grade 5 (fatal) cardiac adverse event by age (≥ 65 vs. < 65 years) and sex (male vs. female) for patients treated with ICIs in 10 trials (Table 1) leading to approvals in the adjuvant setting. *N*, total number of patients in each group.

## 4 Discussion

This analysis represents the largest evaluation of cardiotoxicity associated with immune checkpoint inhibitor (ICI) therapy among patients with curable solid tumors. There are several important findings from the current study. First, our data confirm that immune checkpoint inhibitors are highly efficacious in enhancing cancer‐free survival in the curative setting. Second, multiple types of cardiotoxicities associated with ICIs (Central illustration) were detected, yet the most common toxicity was the development of cardiac arrhythmias. Third, a higher incidence of serious or fatal cardiac adverse events was reported among patients treated with ICI therapy. Finally, but perhaps most relevant to future drug development, none of the trials performed serial cardiac monitoring, such as electrocardiography (ECG), echocardiography, and cardiac biomarkers, suggesting that the true incidence of cardiotoxicity was markedly underestimated. Moreover, in the curative setting, late cardiotoxicity may occur years after trial completion, further limiting our understanding of the risk–benefit balance of ICI therapy.

Patients in these 10 trials were at high risk for disease recurrence but had no evidence of residual cancer at the time of therapy, making the long‐term impact of ICIs on CV risk more relevant. Therapies such as ICIs that can prevent tumor recurrence have become an important advancement; the ICIs, pembrolizumab, atezolizumab, nivolumab, and ipilimumab all decreased the chance of disease recurrence across trials with 3–5 years of follow‐up, some almost doubling recurrence‐free survival (Table 1) (Ascierto et al. 2020; Eggermont et al. 2015; Darvin et al. 2018; Hodi et al. 2010; Kelly et al. 2021; Sharma et al. 2021; Bajorin et al. 2021; Thuny et al. 2022). At the same time, when delivering therapy to patients with potentially curable cancers, the incidence of serious toxicities should be considered carefully, and their impact may only become apparent after trials are completed. With long‐term survival from cancer treatment, the risk of immediate or future CVD with such therapies becomes even more relevant. The possibility that some of these cardiac AEs may not be captured by limited symptom‐based follow‐up monitoring remains an ongoing concern.

The reported incidence of cardiac adverse events with immunotherapy ranges from 3% to 6% (Patel et al. 2023; Piazza et al. 2025; Sharma et al. 2024; Rubio‐Infante et al. 2021; Li et al. 2025, 2022; Nielsen et al. 2024). A meta‐analysis of 51 trials reviewing cardiac immune‐related AEs reported that approximately 3% of patients receiving ICI monotherapy experienced cardiac adverse reactions, with myocarditis occuring in 0.72% (Rubio‐Infante et al. 2021). Other meta‐analyses using public databases have reported higher risks of myocarditis, pericardial disease, and myocardial infarction in patients treated with ICI vs. non‐ICI therapies in the metastatic and early‐stage settings. However, the overall incidence of these cardiac toxicities in individual trials is between 0.04 to 5% (Rubio‐Infante et al. 2021; Li et al. 2025, 2022; Nielsen et al. 2024). In this pooled analysis, the rates of cardiac AEs were similar to those reported previously, with the incidence of overall cardiac AEs approximately 6% and myocarditis reported in 0.4% of patients. In contrast to prior studies, our analysis reports cardiac toxicity data from randomized trials supporting drug approvals and specifically evaluated cardiac adverse events associated with ICIs given for a finite time to treat early‐stage cancer.

The assessment of cardiotoxicity incidence is limited by the absence of systematic, serial cardiac assessments. This precluded capture of asymptomatic cardiac toxicities (e.g., reductions in left ventricular ejection fraction) which could adversely impact development of overt CVD later in life. Routine ECG and physical examinations alone are likely inadequate for capturing the development of early cardiac toxicities. With reliance on patients' symptoms to uncover cardiac adverse events without routine cardiac imaging or surveillance, it is likely that the incidence of AEs such as left ventricular systolic dysfunction will be meaningfully underestimated, since > 50% of patients will either be asymptomatic or go unrecognized (Campbell et al. 2024). Moreover, arrhythmia, an important toxicity reported with ICIs, may not be completely characterized without continuous (e.g., Holter) ECG monitoring. The ten trials included in this analysis did not use the same trial assessments or schedules; there was inconsistency across trials in frequency of cardiac monitoring and parameters to trigger serial cardiac imaging.

Cardiac imaging may be triggered by elevations in serum biomarkers such as N‐terminal pro‐brain natriuretic hormone (NT‐BNP) and cardiac high‐sensitivity troponin. However, trials in this pooled analysis did not capture consistent biomarker data, and more widespread use of these markers has evolved since the initial approvals of ICIs (Ananthan and Lyon 2020). Cardiac biomarkers may be reliable tools in the future to evaluate patients for early signs of cardiac toxicity, though their false positive rate has the potential for increasing the burden on patients for downstream imaging appointments or invasive testing. Current European Society of Cardiology guidelines recommend a risk‐based approach to cardiac monitoring, including biomarkers, during oncology treatment (Ellison and Nohria 2023). In contrast to waiting for AEs to occur before implementing additional monitoring, this approach determines patients' baseline risk of CVD and the specific chemotherapy toxicity attributes and then tailors testing and interventions accordingly. While this may be challenging to implement during a clinical trial, it could help focus additional monitoring on patients who are at greatest risk for long‐term toxicity. Though echocardiograms are increasingly included in trials evaluating treatments with known cardiac risks, there is less consistency with the use of biomarkers and with monitoring of newer therapies with unknown cardiac effects. With emerging data on the long‐term impact of cancer therapies and the use of ICIs in curative or maintenance settings, designing trials with these guidelines in mind, including risk‐based interventions and testing for patients receiving ICIs, could help address some of the gaps in knowledge of cardiac toxicities.

We acknowledge several limitations to our study. Metabolic changes like thyroid disease and hyperlipidemia (Table 3) could contribute to long‐term atherosclerosis risk; however, their long‐term impact remains unclear and cannot be disentangled from the established direct risk of atherosclerosis with ICI treatment (Drobni et al. 2023; Ioannidis 2018). While all patients had surgical procedures prior to randomization, patients in the urothelial and NSCLC studies may have received prior chemotherapy, while other patients in the analysis may not have received any systemic or radiation therapy prior to ICI. Receipt of cardiotoxic therapies such as anthracyclines and/or chest radiation could have increased patients' risk of experiencing CV adverse events; yet these risk factors varied across trials and could not be incorporated in these descriptive analyses. Another limitation is the evolution of guidelines that impact the coding of cardiovascular adverse events. During the study period, the American College of Cardiology/American Heart Association guidelines altered the threshold for diagnosing hypertension in 2017 (Nielsen et al. 2024). Finally, the absence of data on cardiac events beyond 3–5 years contributes to uncertainty regarding the long‐term cardiotoxicity risks. Future efforts to obtain long‐term data through real‐world sources, retrospective studies, and innovations in trial design (i.e., long‐term extension) are warranted.

## 5 Conclusions

Cancer immunotherapy with ICIs has dramatically enhanced survival. This is one of the first analyses exploring cardiotoxicity in patients with solid tumors receiving ICIs for cancer treatment in the curative setting and demonstrates a higher number of serious cardiac AEs, particularly arrhythmia, despite limited to no routine cardiac monitoring. Innovative trial designs, collaborations with providers and patients, and optimization of safety data from clinical trials are priority initiatives at the FDA that may effectuate the goal of increasing cancer survivorship while ensuring protection from cardiotoxiicty (U.S. Food and Drug Administration 2024; Patel et al. 2024).

## Author Contributions

The author takes full responsibility for this article.

## Disclosure

Asma Dilawari‐Reimagine Care Inc. Medical oncology consultant until March 2023, River Fall Associates LLC, Integrative oncology practice. Mori Krantz‐President of Colorado American College of Cardiology. None of the other authors listed have any disclosures.

## Conflicts of Interest

The authors declare no conflicts of interest.

## Data Availability

Primary data for this publication belongs to the companies submitting the respective drug approval applications. Published data is also available for each of the respective trials.
